# Metabolic health is a determining factor for incident colorectal cancer in the obese population: A nationwide population‐based cohort study

**DOI:** 10.1002/cam4.3607

**Published:** 2020-11-20

**Authors:** Yun Kyung Cho, Jiwoo Lee, Hwi Seung Kim, Joong‐Yeol Park, Woo Je Lee, Ye‐Jee Kim, Chang Hee Jung

**Affiliations:** ^1^ Department of Internal Medicine Hallym University Sacred Heart Hospital Hallym University College of Medicine Anyang Republic of Korea; ^2^ Department of Internal Medicine Asan Medical Center University of Ulsan College of Medicine Seoul Republic of Korea; ^3^ Department of Clinical Epidemiology and Biostatistics Asan Medical Center University of Ulsan College of Medicine Seoul Republic of Korea

**Keywords:** colorectal cancer, metabolic syndrome, nationwide cohort study, obesity, phenotypic transition

## Abstract

**Background:**

The association of the risk of colorectal cancer (CRC) with obesity or obesity‐induced metabolic disturbances remains controversial. We assessed the association of metabolic health status with incident CRC among subjects with obesity.

**Methods:**

This study included 319,397 subjects from the Korean National Health Insurance Service‐National Health Screening Cohort. Transitions in metabolic health status and obesity were examined during 2009–2010 and 2011–2012. We categorized subjects with obesity into four separate groups according to their dynamic metabolic health status: metabolically healthy obesity (MHO), MHO to metabolically unhealthy obesity (MUO), MUO to MHO, and stable MUO. Subjects were followed up from 2009 to 2015 for incident CRC.

**Results:**

The stable MHO group showed no increased risk of incident CRC (hazard ratio [HR], 0.97; 95% confidence interval [CI], 0.83–1.14). However, the MHO to MUO group had a higher risk of incident CRC than the stable metabolically healthy nonobese (MHNO) group (HR, 1.34; 95% CI, 1.15–1.57). Among patients with MUO at baseline, those in the subgroup who transitioned to MHO group were not at increased risk of CRC (HR, 1.06; 95% CI, 0.91–1.25), whereas those who remained in the stable MUO group had a higher risk of incident CRC than those in the stable MHNO group (HR, 1.29; 95% CI, 1.19–1.41).

**Conclusions:**

The transition of metabolic health was a determining factor for CRC among subjects with obesity. Hence, maintenance or recovery of metabolic health should be addressed to prevent CRC in individuals with obesity.

## Graphical abstract

The presence of metabolic unhealthiness significantly increased the risk of incident colorectal cancer in the obese population. Physicians should consider the metabolic health status of obese patients and counsel them on the importance of metabolic fitness.

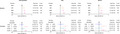

## 1 INTRODUCTION

Obesity is a multifactorial chronic disease that has become a global epidemic over the last few decades.^1^ The implications of obesity including the development of metabolic disorders such as type 2 diabetes, dyslipidemia, and cardiovascular disease (CVD), account for a majority of global health concerns surrounding obesity. Furthermore, the increasing prevalence of obesity is also associated with the incidence of several types of cancer.^2^ Particularly, obesity is a risk factor for colorectal cancer (CRC), which is one of the most common gastrointestinal malignant tumors, associated with high morbidity and mortality worldwide.^3^

Despite the strong epidemiological data on the association between obesity and the increased risk of CRC, underlying mechanisms connecting obesity to CRC remain unclear.^4^ Obesity and metabolic syndrome (MetS) are interrelated conditions that share several pathophysiological mechanisms that appear to accompany each other.^5^ Therefore, the importance of obesity per se as an independent contributor to cardiometabolic disorders, irrespective of obesity‐induced metabolic disturbances, is still uncertain. A subset of obese individuals does not exhibit obesity‐related metabolic derangements such as insulin resistance, hyperglycemia, dyslipidemia, and high blood pressure (BP), despite excessive body fat accumulation.^6^, ^7^, ^8^ This population is referred to as having “metabolically healthy obesity (MHO).”^6^, ^7^, ^8^ Thus far, substantial evidence has demonstrated that patients with MHO have a lower mortality and lower risk of CVD than those with “metabolically unhealthy obesity (MUO),” and they do not have a higher risk of CVD than those with normal weight.^6^, ^7^, ^8^ However, the prognostic value of MHO faces a considerable challenge, and the value may rely on the used health outcomes.^9^, ^10^ Studies have reported conflicting results regarding the risk of CRC in patients with MHO; however, increased risk of CRC has been significantly associated with MUO.^11^, ^12^, ^13^, ^14^, ^15^ Hence, it is unclear whether obesity plays a role in the development of CRC, independent of obesity‐related metabolic disturbances.

Furthermore, metabolic health is a dynamic condition that could change unnoticeably over time in the obese population. For instance, almost 50% of the Multi‐Ethnic Study of Atherosclerosis participants, defined as MHO at baseline, developed metabolic abnormalities during the approximate 12‐year follow‐up period.^16^ In a Korean population‐based prospective cohort study, while 14.5% of the baseline MHO group evolved to MUO status, 29.0% of the baseline MUO group improved to MHO at 4‐year intervals.^17^ Because these phenotypic transitions have been shown to alter the risk of cardiometabolic complications over time,^16^ the previously reported association between obesity and incident CRC should also be carefully interpreted.

Thus, this study was designed to elucidate the impact of the dynamic metabolic health status on the risk of developing CRC among the obese population using a large population‐based cohort from a national health screening examination.

## 2 MATERIALS AND METHODS

### 2.1 Study population

We gathered data from the Korean National Health Insurance Service‐National Health Screening Cohort (NHIS‐HEALS). Currently, the Korean National Health Insurance Service (NHIS) is in charge of maintaining and managing databases regarding all health services use throughout Korea.^18^ A random sample of 514,866 subjects included in the present study represents about 10% of the total participants in NHIS health screening examinations from 2002 to 2003. Follow‐up examinations were performed until emigration, death, or end of the study period in 2015, whichever occurred first.^18^ This cohort data include information on general health examinations of people who underwent biannual examinations. Further information on the Korean NHIS‐HEALS has been described previously.^18^

The demographic and biochemical data of the cohort from 1 January 2009 to 31 December 2010 were used. This 2‐year period was defined as the index period because from 2009, the NHIS‐HEALS began to include several laboratory data in the examination, such as triglyceride (TG) and high‐density lipoprotein cholesterol (HDL‐C) levels, necessary in defining the baseline metabolic health of the subjects.^18^ Then, the next biannual health examination data from 1 January 2011 to 31 December 2012 were used to examine the changes in metabolic health status and obesity during that period.

Of the 514,866 people assessed in the NHIS‐HEALS, we excluded those who died (*n* = 24,593) or had a history of CRC (*n* = 57,192), other malignant neoplasia (*n* = 22,740), and/or colon or rectal polyps (*n* = 38,456) before the end of the index period. Participants having missing values for baseline body mass index (BMI), systolic BP, diastolic BP, fasting plasma glucose (FPG), TG, and HDL‐C were also excluded. Hence, 319,397 people were included in our analysis. When the transition in obesity and metabolic health was considered, we analyzed only the stable obese population to minimize any possible effect of incident CRC on body weight changes.

This study was approved by the NHIS inquiry commission. Informed consent was not obtained from each participant because the current study was based on the NHIS‐HEALS results, and all data were de‐identified and fully anonymized for all analyses. This study was approved by the Institutional Review Board of Asan Medical Center (IRB No. 2020‐0491).

### 2.2 Definitions of metabolic health and obesity states

We used Asia‐Pacific criteria to define obesity (BMI ≥ 25 kg/m^2^) and non‐obesity (BMI < 25 kg/m^2^). The criteria was validated by the World Health Organization Western Pacific Region,^19^ the Korean Centers for Disease Control and Prevention, and the Korean Society for the Study of Obesity.^20^, ^21^ The Adult Treatment Panel III criteria were adopted to define metabolic health as having none or one of the following risk factors^22^: (1) systolic BP ≥130 mmHg and/or diastolic BP ≥85 mmHg and/or taking antihypertensive medications; (2) TG level ≥150 mg/dl and/or taking lipid‐lowering medications; (3) FPG level ≥100 mg/dl and/or taking antidiabetic medications; and (4) HDL‐C levels <40 mg/dl in men and <50 mg/dl in women. Based on these criteria, all study subjects were labeled as one of the following groups: (1) metabolically healthy, nonobese (MHNO), with BMI < 25 kg/m^2^ and no or one metabolic risk factor; (2) metabolically unhealthy, nonobese (MUNO), with BMI < 25 kg/m^2^ and ≥2 metabolic risk factors; (3) MHO, defined as BMI ≥ 25 kg/m^2^ and no or one metabolic risk factor; and (4) MUO, with a BMI ≥ 25 kg/m^2^and ≥2 metabolic risk factors.

Participants were further categorized into the following four groups which reflected their metabolic health transition in terms of obesity at the follow‐up biannual health examination between 2011 and 2012: stable MHO, MHO to MUO, MUO to MHO, and stable MUO groups.

### 2.3 Definitions of CRC and metabolic comorbidities

The presence of CRC was identified by International Classification of Diseases, 10th Revision (ICD‐10) codes C18–C20 (malignant neoplasm of the colon, rectosigmoid junction, or rectum) or D010–D012 (carcinoma in situ/intramucosal adenocarcinoma). Colon and rectal polyps were also defined by ICD‐10 codes K63.5 (polyp of colon) and K62.1 (rectal polyp), respectively.

The following criteria were used for defining patients with type 2 diabetes: (1) having antidiabetic drugs; and (2) ICD‐10 codes for noninsulin‐dependent diabetes mellitus (E11), malnutrition‐related diabetes mellitus (E12), other specified diabetes mellitus (E13), or unspecified diabetes mellitus (E14) as either a principal or secondary diagnosis. We regarded patients as those with hypertension if they had antihypertensive medications and ICD‐10 codes for essential (primary) hypertension (I10), hypertensive heart disease (I11), hypertensive chronic kidney disease (CKD) (I12), hypertensive heart and CKD (I13), or secondary hypertension (I15) as either a principal or secondary diagnosis. Patients with dyslipidemia were identified by the operation condition of both having lipid‐lowering drugs and the ICD‐10 code for disorders of lipoprotein metabolism and other lipidemia (E78) as either a primary or secondary diagnosis.

### 2.4 Covariates

Age, sex, income, smoking habits (current smoker, ex‐smoker, or nonsmoker), drinking habits (heavy drinking, moderate, mild, or none), and presence of inflammatory bowel disease (IBD) in the baseline health examination were included as covariates. Heavy drinkers were defined as those consuming ≥7 drinks on the same occasion and drinking >5 days per week, while mild and moderate drinkers were those consuming <7 drinks on any single day and who drank 1–2 or 3–4 days per week, respectively. Patients having IBD were defined as those who met all of the following three criteria: (1) diagnostic codes for Crohn's disease (CD, K50.0‒50.9) or ulcerative colitis (UC, K51.0–51.9) as the primary or subsidiary diagnosis, (2) prescribed medications for CD or UC, and (3) rare intractable disease registration code for CD (V130) or UC (V131).

### 2.5 Statistical analyses

Continuous data are expressed as means ± standard deviations, and categorical data are expressed as percentages. Analysis of variance and Scheffe's test for post hoc analysis or the chi‐square test were used for comparing the baseline characteristics of the subjects regarding their metabolic health and obesity status. We conducted a multiple imputation procedure with a fully conditional specification method to impute missing values for smoking, drinking, and physical activities. The five imputed data sets were created with 20 burn‐in iterations, analyzed by the same analytical procedures, and the results from these analyses were combined to obtain an overall estimate.

Cox proportional hazard analyses were performed to estimate the hazard ratio (HR) and 95% confidence interval (CI) of incident CRC during the follow‐up period. Multivariable models were adjusted for age, sex, income, smoking, and alcohol drinking and presence of IBD. We evaluated the risk of incident CRC with a fixed set of covariates adjusted with significant results in univariate analysis and variables considered clinically important. The results of univariate analysis are presented in Table S1. The risk of incident CRC was first analyzed according to the baseline metabolic health and obesity without considering their transition in reference to the MHNO group. Next, the risk was further assessed after considering the transition of metabolic health and obesity in subjects with obesity at baseline. The stable MHNO group during the follow‐up period was considered the reference group. Because previous studies have repeatedly reported sex differences in the association between the risk of CRC and obesity status,^4^ we also performed a sex‐specific analysis. All *p*‐values were two‐tailed, with statistical significance set at *p* < 0.05. All statistical analyses were performed using SAS Enterprise Guide software, Version 7.1 (SAS Institute, Inc.).

## 3 RESULTS

### 3.1 Baseline characteristics of the entire patients

Table 1 shows the clinical and biochemical characteristics of the patients stratified according to BMI categories and metabolic health status at baseline. The prevalence of MHNO, MHO, MUNO, and MUO was 29.3% (*n* = 93,577), 8.9% (*n* = 28,577), 34.8% (*n* = 111,025), and 27.0% (*n* = 86,238), respectively. Compared with the healthy and lean patients, the patients with MHO were characterized by worse lipid profile which were elevated TG, LDL‐C, and total cholesterol levels and reduced HDL‐C level. In contrast, subjects with MHO exhibited more favorable risk profiles than those with MUNO or MUO. FPG and TG levels were lower and HDL‐C levels were higher in the MHO group than in the MUNO group. Among the study patients, more male patients were categorized into the unhealthy groups (i.e., MUNO and MUO groups).

**TABLE 1:** Characteristics of study participants according to baseline metabolic health and obesity status.

Baseline category BMI Metabolic health status	MHNO <25 kg/m^2^ 0–1 risk factor	MHO ≥25 kg/m^2^ 0–1 risk factor	MUNO <25 kg/m^2^ ≥2 risk factors	MUO ≥25 kg/m^2^ ≥2 risk factors	*p*‐value
**N**	93,577 (29.3)	28,557 (8.9)	111,025 (34.8)	86,238 (27.0)	
Sex (% men)	47.2	48.4	54.4	56.3	<0.0001
Age (year)	57.5 ± 8.6^a^	57.5 ± 8.0^a^	60.3 ± 9.2	59.3 ± 8.5	<0.0001
BMI (kg/m^2^)	22.1 ± 1.9	26.8 ± 1.7	22.7 ± 1.7	27.2 ± 2.0	<0.0001
WC (cm)	76.9 ± 6.7	86.8 ± 6.4	80.3 ± 6.4	89.4 ± 6.6	<0.0001
Systolic BP (mmHg)	119.2 ± 14.1	123.5 ± 14.3	129.9 ± 14.7	132.4 ± 14.5	<0.0001
Diastolic BP (mmHg)	74.0 ± 9.3	76.5 ± 9.6	80.4 ± 9.5	82.1 ± 9.6	<0.0001
Smoking (%)					
Current smoker	15.9	12.7	19.9	17.5	<0.0001
Ex‐smoker	14.3	16.8	17.4	20.7	
Nonsmoker	67.1	67.3	59.6	58.7	
Drinking (%)					
None	60.8	59.1	56.6	54.1	<0.0001
Mild	18.5	17.5	16.1	15.8	
Moderate	4.0	3.9	4.7	4.1	
Heavy	14.6	17.1	20.2	23.5	
Physical activity (%)					
None	27.2	27.9	30.0	30.0	<.0001
1–2 times/week	22.3	22.8	21.7	23.3	
3–4 times/week	21.3	20.6	20.3	20.3	
≥5 times/week	27.5	26.6	25.9	24.3	
Medical history (%)					
Type 2 diabetes	1.2	1.1	15.7	18.4	<0.0001
HTN	13.1	22.1	44.7	55.8	<0.0001
Dyslipidemia	3.8	4.1	29.4	34.8	<0.0001
IBD	0.08	0.04	0.05	0.05	0.006
Income					
Medicaid	0.2	0.2	0.3	0.3	<0.0001
First quintiles (lowest)	13.4	13.5	14.6	14.3	
Second quintiles	13.7	12.9	13.7	13.2	
Third quintiles	16.1	16.6	16.2	15.9	
Fourth quintiles	20.3	20.7	21.2	21.8	
Fifth quintiles (highest)	36.4	36.0	34.0	34.6	
FPG (mg/dl)	91.6 ± 12.6	92.3 ± 12.2	108.7 ± 30.2	110.7 ± 30.0	<0.0001
TG (mg/dl)	97.8 ± 46.2	109.0 ± 49.3	167.0 ± 102.2	189.1 ± 111.8	<0.0001
LDL‐C (mg/dl)	120.2 ± 33.9	126.1 ± 33.9	121.4 ± 41.0	122.9 ± 42.4	<0.0001
HDL‐C (mg/dl)	59.6 ± 25.5	57.0 ± 24.0	51.6 ± 27.0	49.3 ± 24.2	<0.0001
TC (mg/dl)	198.7 ± 33.2	204.1 ± 33.7^a^	204.1 ± 39.9^a^	207.4 ± 40.0	<0.0001

Results reported as means ± standard deviations, unless otherwise indicated.

All variables were statistically different among the four groups, unless otherwise indicated.

Abbreviations: BMI, body mass index; BP, blood pressure; eGFR, estimated glomerular filtration rate; FPG, fasting plasma glucose; HDL‐C, high‐density lipoprotein cholesterol; HTN, hypertension; IBD, inflammatory bowel disease; LDL‐C, low‐density lipoprotein cholesterol; MHNO, metabolically healthy non‐obesity; MHO, metabolically healthy obesity; MUNO, metabolically unhealthy obesity; MUO, metabolically unhealthy obesity; TC, total cholesterol; TG, triglyceride; WC, waist circumference.

^a^ No statistical difference was observed.

### 3.2 Prognosis associated with baseline metabolic health and obesity state

Table 2 shows the risk for incident CRC according to baseline metabolic health and obesity without considering the transition over time. In the total population, the risk of incident CRC in the obese groups, irrespective of the metabolic health status, was significantly higher than that in the MHNO group (multivariable‐adjusted HR, 1.14; 95% CI, 1.04–1.26 in the MHO group vs. multivariable‐adjusted HR 1.21; 95% CI, 1.13–1.29 in the MUO group). The risk of incident CRC in the MUNO group was also significantly higher than that in the lean, healthy population, with a multivariable‐adjusted HR of 1.19 (95% CI, 1.12–1.27).

**TABLE 2:** Risk of incident CRC according to baseline metabolic health and obesity status.

Total population				
Baseline category BMI Metabolic health status	MHNO <25 kg/m^2^ 0–1 risk factor	MHO ≥25 kg/m^2^ 0–1 risk factor	MUNO <25 kg/m^2^ ≥2 risk factors	MUO ≥25 kg/m^2^ ≥2 risk factors
Total population				
N (% of total)	93,577 (29.3)	28,557 (8.9)	111,025 (34.8)	86,238 (27.0)
Number of events (%)	1643 (1.8)	567 (2.0)	2644 (2.4)	2009 (2.3)
Crude HR (95% CI)	1 (ref)	1.13 (1.03–1.24)	1.36 (1.28–1.45)	1.32 (1.24–1.41)
*p*‐value	Ref	0.013	<0.001	<0.001
Multivariable‐adjusted HR (95% CI)^a^	1 (ref)	1.14 (1.04–1.26)	1.19 (1.12–1.27)	1.21 (1.13–1.29)
Multivariable‐adjusted *p*‐value	Ref	0.006	<0.001	<0.001
Men				
N (% of total)	44,194 (26.5)	13,824 (8.3)	60,416 (36.2)	48,536 (29.1)
Number of events (%)	935 (2.1)	314 (2.3)	1625 (2.7)	1262 (2.6)
Crude HR (95% CI)	1 (ref)	1.08 (0.95–1.23)	1.28 (1.18–1.38)	1.23 (1.13–1.34)
*p*‐value	Ref	0.250	<0.001	<0.001
Multivariable‐adjusted HR (95% CI)^b^	1 (ref)	1.18 (1.04–1.34)	1.23 (1.13–1.33)	1.29 (1.19–1.41)
Multivariable‐adjusted *p*‐value	Ref	0.011	<0.001	<0.001
Women				
N (% of total)	49,383 (32.4)	14,733 (9.7)	50,609 (33.2)	37,702 (24.7)
Number of events (%)	708 (1.4)	253 (1.7)	1019 (2.0)	747 (2.0)
Crude HR (95% CI)	1 (ref)	1.19 (1.03–1.37)	1.40 (1.27–1.54)	1.36 (1.23–1.51)
*p*‐value	Ref	0.019	<0.001	<0.001
Multivariable‐adjusted HR (95% CI)^b^	1 (ref)	1.14 (0.99–1.32)	1.21 (1.09–1.33)	1.17 (1.06–1.30)
Multivariable‐adjusted *p*‐value	Ref	0.066	<0.001	<0.001

Abbreviations: CI, confidence interval; CRC, colorectal cancer; HR, hazard ratio; MHNO, metabolically healthy non‐obesity; MHO, metabolically healthy obesity; MUNO, metabolically unhealthy non‐obesity; MUO, metabolically unhealthy obesity.

^a^ Adjusted for baseline age, sex, income, smoking, alcohol drinking, and presence of IBD.

^b^ Adjusted for baseline age, income, smoking, alcohol drinking, and presence of IBD.

In subgroup analyses according to sex, the risk of incident CRC in the respective groups showed similar trends to those in the total population. The risk of incident CRC in the MHO group, MUNO group, and MUO group was higher than that in the MHNO group, with multivariable‐adjusted HRs of 1.18 (95% CI, 1.04–1.34), 1.23 (95% CI, 1.13–1.33), and 1.29 (95% CI, 1.19–1.41), respectively (Table 2). However, in women, the MHO status did not confer a significantly increased risk of incident CRC compared with the MHNO status (multivariable‐adjusted HR, 1.14; 95% CI, 0.99–1.32). Similar to men, the MUNO and MUO groups had higher risks of incident CRC than the MHNO group, with multivariable‐adjusted HRs of 1.21 (95% CI, 1.09–1.33) and HR 1.17 (95% CI, 1.06–1.30), respectively (Table 2).

### 3.3 Changes in the metabolic health status of the obese patients and their risk of incident CRC

Among those with MHO at baseline, 47.3% remained in the MHO group (stable MHO), while 35.3% were still obese and transitioned to a metabolically unhealthy status (MHO to MUO). In the baseline MUO group, 7.6% recovered to a metabolically healthy status (MUO to MHO) and 40.5% remained in the MUO group (stable MUO).

**FIGURE 1:**
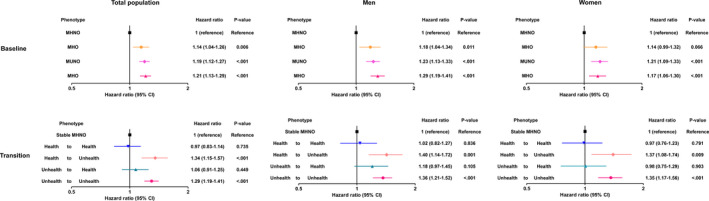
The risk of incident CRC (A) without and (B) with consideration of transition in metabolic health status in the obese population. CRC, colorectal cancer; MHNO, metabolically healthy non‐obesity; MHO, stable metabolically healthy obesity; MUNO, metabolically unhealthy non‐obesity; MUO, metabolically unhealthy obesity.

Table 3 shows the crude and adjusted HRs for incident CRC of the obese population regarding the stable MHNO group as the reference and considering the status transitions. In comparison with the stable MHNO group, the stable MUO group had a significantly higher risk of incident CRC (multivariable‐adjusted HR, 1.29; 95% CI, 1.19–1.41). The risk of CRC development was much higher in people who progressed to the MHO to MUO group than in those in the reference group, despite their metabolic health at baseline, with a multivariable‐adjusted HR of 1.34 (95% CI: 1.15–1.57). In contrast, the risk of incident CRC was not increased in the stable MHO group (multivariable‐adjusted HR, 0.97; 95% CI, 0.83–1.14) and the MUO to MHO group (multivariable‐adjusted HR, 1.06; 95% CI, 0.91–1.25). Subgroup analyses according to sex showed the same pattern in men and women, with an increased risk of incident CRC in the MHO to MUO and stable MUO groups and no increase in the risk of incident CRC in the stable MHO group and MUO to MHO group (Table 3). The multivariable‐adjusted HRs for incident CRC for the total population, men, and women are shown in Figure 1.

**TABLE 3:** Risks of incident CRC according to the transition from metabolically healthy to unhealthy status among subjects with obesity in reference to the stable MHNO group.

Baseline obesity status	MHNO	MHO		MUO	
Follow‐up category	MHNO	MHO Stable healthy	MUO Healthy to unhealthy	MHO Unhealthy to healthy	MUO Stable unhealthy
Total population					
N (% of respective baseline category)	56,157 (69.3)	11,641 (47.3)	8715 (35.3)	9914 (7.6)	53,073 (40.7)
Number of events (%)	901 (1.60)	179 (1.54)	199 (2.28)	179 (1.81)	1246 (2.35)
Crude HR (95% CI)	1 (ref)	0.96 (0.82–1.13)	1.42 (1.22–1.66)	1.20 (0.95–1.31)	1.46 (1.34–1.59)
*p*‐value	Ref	0.609	<0.001	0.169	<0.001
Multivariable‐adjusted HR (95% CI)^a^	1 (ref)	0.97 (0.83–1.14)	1.34 (1.15–1.57)	1.06 (0.91–1.25)	1.29 (1.19–1.41)
Multivariable‐adjusted *p*‐value	Ref	0.735	<0.001	0.449	<0.001
Men					
N (% of respective baseline category)	25,515 (66.9)	5507 (72.4)	4382 (17.8)	5565 (13.2)	30,522 (72.2)
Number of events (%)	504 (1.98)	99 (1.80)	114 (2.60)	118 (2.12)	783 (2.57)
Crude HR (95% CI)	1 (ref)	0.92 (0.74–1.14)	1.33 (1.09–1.63)	1.08 (0.88–1.32)	1.31 (1.17–1.46)
*p*‐value	Ref	0.439	0.006	0.454	<0.001
Multivariable‐adjusted HR (95% CI)^b^	1 (ref)	1.02 (0.82–1.27)	1.40 (1.14–1.72)	1.18 (0.97–1.45)	1.36 (1.21–1.52)
Multivariable‐adjusted *p*‐value	Ref	0.836	0.001	0.105	<0.001
Women					
N (% of respective baseline category)	30,642 (71.4)	6.134 (48.3)	4333 (17.6)	4349 (13.6)	22,551 (70.4)
Number of events (%)	397 (1.30)	80 (1.30)	85 (1.96)	61 (1.40)	463 (2.05)
Crude HR (95% CI)	1 (ref)	1.00 (0.79–1.27)	1.49 (1.18–1.88)	1.06 (0.81–1.38)	1.31 (1.17–1.46)
*p*‐value	Ref	0.993	<0.001	0.693	<0.001
Multivariable‐adjusted HR (95% CI)^b^	1 (ref)	0.97 (0.76–1.23)	1.37 (1.08–1.74)	0.98 (0.75–1.29)	1.35 (1.17–1.56)
Multivariable‐adjusted *p*‐value	Ref	0.791	0.009	0.903	<0.001

Abbreviations: CI, confidence interval; CRC, colorectal cancer; HR, hazard ratio; MHNO, metabolically healthy non‐obesity; MHO, metabolically healthy obesity; MUNO, metabolically unhealthy non‐obesity; MUO, metabolically unhealthy obesity.

^a^ Adjusted for baseline age, sex, income, smoking, alcohol drinking, and presence of IBD.

^b^ Adjusted for baseline age, income, smoking, alcohol drinking, and presence of IBD.

## 4 DISCUSSION

The present study showed that metabolic unhealthiness significantly contributed to the incident CRC in the obese population. When the transition of metabolic health was considered, maintenance or recovery of metabolic health alleviated the risk of CRC, even if the patients were still obese. A consistent metabolic unhealthiness or transition to metabolic unhealthy status significantly increased the risk of CRC in the obese population. Our results show that metabolic unhealthiness and not obesity is a crucial risk factor for CRC.

MetS describes a cluster of metabolic abnormalities characterized by insulin resistance, with pro‐inflammatory changes and a predisposition to type 2 diabetes, dyslipidemia, and premature atherosclerosis, which is a risk factor of CRC.^4^, ^23^ In the MetS and cancer project, MetS was associated with an increased risk of CRC.^24^ Given this close association between MetS and CRC, metabolic disturbances induced by obesity have been suggested as a mediator for the association between obesity and CRC.^4^ Although obesity frequently leads to the development of MetS, obesity is a highly heterogeneous condition and not every obese patient has obesity‐related metabolic comorbidities or MetS.^25^ Therefore, we hypothesized that if the effect of obesity on CRC had a dependent effect on metabolic deterioration, metabolic health status would be a determining factor for CRC incidence in subjects with obesity. In the present analysis, we observed that the risk of CRC incidence among patients with obesity was highly dependent on the metabolic health status. Specifically, the risk of incident CRC was high in people who had progressed to a metabolic unhealthy status and in those who maintained a constant MUO status (Table 3 and Figure 1). People who maintained metabolic health or recovered their metabolic health from an unhealthy state were not at a higher risk for developing CRC, despite still being obese (Table 3 and Figure 1). Collectively, these findings suggest that metabolic health is the determining factor for incident CRC in subjects with obesity.

So far, limited longitudinal studies have investigated the association between obesity and metabolic health in term of risks of developing CRC. In a case‐control study by Murphy et al., based on the European Prospective Investigation into Cancer and Nutrition study, a higher CRC risk was observed among metabolically unhealthy/normal weight and metabolically unhealthy/overweight (BMI ≥ 25 kg/m^2^) participants but not among metabolically healthy/overweight individuals.^11^ A retrospective cross‐sectional study of 70,428 individuals in Korea who underwent colonoscopy showed that obesity increased the risk of colorectal neoplasms (CRNs), including adenomas and that a metabolically unhealthy status added to that risk. However, for advanced cancer, a metabolically unhealthy status increased the risk of CRC, while obesity did not.^13^ These results suggest metabolic unhealthiness, rather than obesity, affects the incidence and development of CRC. However, previous reports have limitations, as they were derived from the static condition of MHO.

Previous studies have reported that one‐third to one‐half of people with MHO transitioned to a metabolically unhealthy state, while one‐fourth to one‐third of patients with MUO recovered their metabolic health.^17^, ^26^, ^27^, ^28^, ^29^ This transition may alter the risk of examined health, including CRC, over time. Recently, given the concept that metabolic health could be transient, researchers have tried new approaches to examine the effect of metabolic health on various outcomes by assessing its transitions. Kim et al. observed that metabolically healthy status was transient and maintaining metabolic health was critical for the prevention of type 2 diabetes, irrespective of the presence of obesity.^30^ Nam et al. reported that among patients with MHO, those who transitioned to MUO were at a 4.1‐fold increased risk in incident CKD than those who regressed to MHNO.^31^ Recently, we have also demonstrated that the phenotypic changes in MHO affected the risk of cardiovascular events and CKD as well as all‐cause mortality.^26^, ^27^ The present study has elucidated the implications of metabolic health on incident CRC by assessing the status changes among subjects with obesity. This approach showed that a transition to a metabolically unhealthy status was a predictor of poor prognosis and that control of metabolic fitness might be a feasible therapeutic strategy to help prevent CRC in subjects with obesity.

The pathophysiology regarding the link between obesity and increased CRC risk is not fully understood. Chronic inflammation and disturbance of adipokines or growth factors in obesity have been proposed as potential mechanisms.^4^ Recently, Ko et al. reported that MHO increased the risk of CRN including adenoma; however, MHO was not a risk factor for advanced CRN.^14^ In contrast, metabolically unhealthy states (MUNO or MUO) were significantly associated with an increased risk of advanced CRN.^14^ Based on these findings, the authors proposed that obesity alone is not able to promote the progression of CRC, while obesity affects early steps in the adenoma–carcinoma pathway via low‐grade inflammation.^14^ They also inferred that a metabolically unhealthy status might be the next step in the process of colorectal carcinogenesis via increased growth factors (i.e., insulin‐like growth factor or epidermal growth factor receptor) by insulin resistance, which leads to advanced cancer.^14^ Our results did not provide information on the mechanism associated with increased CRC in subjects with obesity. However, our data provide obvious evidence that metabolic unhealthiness is a powerful link between obesity and the incidence of CRC. Clinically, the risk for CRC could be mitigated if patients with obesity maintained or recovered their metabolic health. In other words, metabolic health could a modifiable risk factor in preventing CRC development in the obese population.

Previous studies have suggested that obesity is associated with an increased risk of CRC.^4^ However, dichotomizing results according to sex have been reported.^4^ While the incidence of CRC is consistently and significantly greater in men with obesity, the association between obesity and CRC is weaker among women.^4^, ^32^, ^33^ In addition, a large‐prospective cohort study in Korea has recently reported that obesity (≥25 kg/m^2^) is a risk factor for CRC in men but not in women.^15^ Although the mechanisms still remain unclear, this sex difference may be partially explained by differences in prevalence and age of onset of MetS; there may also be a protective effect of estrogen against atherosclerosis.^4^, ^34^ In the present study, we observed that women in the MHO group did not develop CRC. Our finding, therefore, supports previous reports and further suggests that, at least in women with obesity, combined metabolic unhealthiness is a crucial factor in colorectal carcinogenesis.

This study has some limitations that should be considered in its interpretation. First, there could be selection bias because our study population consisted of those who underwent health examinations; however, this limitation is inevitable in observational studies. Second, because we used claims data, the identification of CRC might not have been completely precise. Furthermore, the stages of CRC were not considered in the analysis. Finally, the current study may not have enough statistical power to fully assess interactions due to a relatively short follow‐up duration. Despite these limitations, this study had strengths in that we analyzed data from a large number of patients using a national cohort sample and elucidated the implications of dynamic metabolic health on incident CRC among subjects with obesity. Our approach showed that transition to a metabolically unhealthy status was a predictor of poor prognosis, and therefore, maintenance or recovery of metabolic health should be embraced to help prevent CRC in people with obesity.

In conclusion, our results were heterogeneous in terms of identifying which patients with obesity had an increased CRC risk according to their metabolic health and that metabolic health was not static. Hence, when evaluating the association between obesity and CRC, clinicians should consider the metabolic health status of patients and counsel them accordingly on the importance of metabolic fitness.

## CONFLICT OF INTEREST

The authors declare no conflict of interest.

## AUTHORS’ CONTRIBUTIONS

Chang Hee Jung: Conceptualization. Ye‐Jee Kim: Data curation and formal analysis. Yun Kyung Cho: Interpretation of the data and writing‐original draft. Jiwoo Lee, Hwi Seung Kim, Joong‐Yeol Park, and Woo Je Lee: writing – review and editing.

## Supporting information

Table S1

## Data Availability

The data that support the findings of this study are available from the corresponding author upon reasonable request.
