# Supplementary material for: Metabolic health is a determining factor for incident colorectal cancer in the obese population: A nationwide population‐based cohort study
Source: Cancer Med. 2020 Nov 20;10(1):220–9. doi: 10.1002/cam4.3607 (PMC7826459; doi:10.1002/cam4.3607)
Supplement: Supplementary file 1 — Table S1 [file CAM4-10-220-s001.docx]

**Table S1.** Univariate analysis for the development of CRC.

| **Variables** |  | **HR** | **95% CI** | **p-value** |
| --- | --- | --- | --- | --- |
| BMI | <25 | 1.000 | Ref | 0.008 |
|  | ≥25 | 1.068 | (1.017 – 1.121) |  |
| Risk factor | 0 | 1.000 | Ref | <.001 |
|  | 1 | 1.146 | (1.045 – 1.258) | 0.004 |
|  | 2 | 1.375 | (1.258 – 1.503) | <.001 |
|  | 3 | 1.488 | (1.359 – 1.630) | <.001 |
|  | 4 | 1.529 | (1.365 – 1.713) | <.001 |
| Gender | Male | 1.000 | Ref | <.001 |
|  | Female | 0.700 | (0.667 – 0.734) |  |
| Age | 40–49 | 1.000 | Ref | <.001 |
|  | 50–59 | 1.354 | (1.231 – 1.489) |  |
|  | 60–69 | 2.100 | (1.909 – 2.310) |  |
|  | 70–79 | 2.917 | (2.641 – 3.221) |  |
|  | 80+ | 3.317 | (2.834 – 3.882) |  |
| Health insurance type | Health insurance | 1.000 | Ref | 0.387 |

|  | Medicaid | 1.466 | (0.974 – 2.205) |  |
| --- | --- | --- | --- | --- |
| Income | Medicaid | 1.473 | (0.978 – 2.220) | 0.064 |
|  | 1st quintiles | 1.044 | (0.970 – 1.125) | 0.251 |
|  | 2nd quintiles | 1.013 | (0.939 – 1.092) | 0.744 |
|  | 3rd quintiles | 0.993 | (0.925 – 1.067) | 0.852 |
|  | 4th quintiles | 0.992 | (0.929 – 1.060) | 0.821 |
|  | 5th quintiles (top) | 1.000 | Ref | 0.379 |
| Smoking | Missing | 1.316 | (1.152 – 1.504) | <.001 |
|  | Non– | 1.000 | Ref | <.001 |
|  | Ex– | 1.301 | (1.224 – 1.384) | <.001 |
|  | Current | 1.297 | (1.220 – 1.380) | <.001 |
| Alcohol intake | Missing | 1.219 | (1.051 – 1.415) | 0.009 |
|  | None | 1.000 | Ref | <.001 |

|  | Mild | 1.005 | (0.939 – 1.074) | 0.893 |
| --- | --- | --- | --- | --- |
|  | Moderate | 1.200 | (1.072 – 1.343) | 0.002 |
|  | Heavy | 1.212 | (1.141 – 1.287) | <.001 |
| Physical activity | Missing | 1.172 | (0.995 – 1.380) | 0.057 |
|  | None | 1.000 | Ref | 0.899 |

|  | 1–2 times/week | 0.888 | (0.830 – 0.951) | 0.001 |
| --- | --- | --- | --- | --- |
|  | 3–4 times/week | 0.983 | (0.919 – 1.052) | 0.627 |
|  | ≥ 5 times/week | 0.978 | (0.918 – 1.042) | 0.491 |
| Serum cholesterol | Baseline LDL-C | 0.998 | (0.997 – 0.998) | <.001 |
|  | Baseline TC | 0.998 | (0.998 – 0.999) | <.001 |
| Presence of disease | Diabetes | 1.445 | (1.352 – 1.545) | <.001 |
|  | HTN | 1.459 | (1.392 – 1.530) | <.001 |
|  | Dyslipidemia | 1.259 | (1.192 – 1.330) | <.001 |
|  | IBD | 1.604 | (0.723 – 3.561) | 0.245 |

**Abbreviations:** BMI, body mass index; HTN, hypertension; IBD, inflammatory bowel disease; LDL-C, low-density lipoprotein cholesterol; TC, total cholesterol;
